# X-Ray in War Surgery

**Published:** 1919

**Authors:** 


***X-Ray in War Surgery.*** By Lieut. Ralph Boerne Bettman, U. S. M. C. (Fort Riley, Kansas, Base Hospital). *Mississippi Valley Medical Journal*, November 1918.

The scope of the X-ray in war surgery is so great that the author confines himself to the use of the X-ray in locating and removing a foreign body.

In locating bullets, large portions of the anatomy must be carefully searched, as a bullet entering the human body may follow an unexpectedly long and deviating course. For this reason it is best to use the fluoroscope in searching for bullets. The author employs the usual average strength generator, tubes, and average fluorescent screen. A little care must be taken; the part observed must be rotated or the tube moved; otherwise small particles hidden under heavy bones may be overlooked. In doubtful cases (small objects hidden in dense tissues) where approximate location is known, it is advisable to take a picture.

For fluoroscope work the operator and the patient are protected by encasing the tube of the container in a lead chamber, and by making use of a one millimeter aluminum screen over the diaphragm.

When a foreign body is found, it must be located as accurately as possible to determine the advisability of operation and the best manner of procedure. While this may be done by usual method of Roentgenograms taken in two directions, the author prefers the fluoroscope for this work as well. He has found the method described by Holzknecht the most satisfactory.

The removal of the bullet after it has been localized is often a difficult task. The most careful localization may be "dislocated" by changes in conditions in the operating room as compared with the conditions during the fluoroscopic examination. The author has devised an apparatus by which the fluoroscope may be used during operation either in daylight or intense artificial light.

This apparatus, which the author calls "cryptoscope" consists of a light-tight box in the shape of a truncated pyramid, the upper end being fastened by straps over the operator's eyes. The base consists of an ordinary fluorescent screen, and when it is closed the operator is looking down in a dark, light-tight box upon the fluorescent screen. The base is on a hinge and may be released by pressing on a button, so that it flies up, and the operator sees the field unimpeded. A piece of lead glass at the base of the pyramid gives further protection.

The cryptoscope may be worn by the assistant. Seeing the bullet, he points to it with an artery snap or probe, and he further aids the surgeon by telling him the relative position of the shadow of the knife, and the shadow of the bullet. When the bullet moves with every slight motion of the knife, it is evident that the two are in close proximity. The cryptoscope could not be used continuously; intermittent use is all that is necessary.

The surgeon may also wear the cryptoscope, keeping the base open mostly, but closing it from time to time to determine the position of the bullet in relation to the probe or forceps held in the wound. Long incisions and unnecessary laceration of tissue are prevented by this method.

